# Supplementary material for: netMUG: a novel network-guided multi-view clustering workflow for dissecting genetic and facial heterogeneity
Source: Front Genet. 2023 Dec 6;14:1286800. doi: 10.3389/fgene.2023.1286800 (PMC10731261; doi:10.3389/fgene.2023.1286800)
Supplement: Supplementary file 1 [file DataSheet1.DOCX]

Supplementary Material

**Data pre-processing steps in the case study**

As mentioned in previous work (White *et al.*, 2021), participants were genotyped with either Illumina or 23andMe arrays, which were subsequently filtered via standard quality control, phased, and imputed to the 1000 Genomes (1000G) Project Phase 3 reference panel. Imputations with low quality (on both the SNP and individual levels) were neglected. SNPs with low minor allele frequency (<0.01) and deviation from Hardy-Weinberg equilibrium (<1e-6) were removed, resulting in 7 141 882 SNPs. The European participants were obtained by finding the nearest neighbors of each individual from the 1000G samples and selecting individuals whose closest neighbors are CEU, TSI, FIN, GBR, or IBS. The face of every individual was scanned as a 3D mesh surface and registered via the MeshMonk framework. Subsequently, facial images were cleaned and mapped to an anthropometric mask with 7160 landmarks. Every face was made symmetric along the X-axis. Outlier images were either removed or re-processed.

We further imputed missing SNPs by the mode and filtered out SNPs with moderate to high linkage disequilibrium (LD) (r­^2^>0.2), resulting in 265 277 SNPs. No additional pre-processing was done on facial data. The population structure of our genomic data was derived through ancestry axes obtained by projecting the data onto the principal component (PC) space of the SNPs from the 1000G Project. To deal with potential confounders in downstream analyses, genomic and facial features were corrected for sex, age, age squared, face size, camera system, and the first four ancestry axes via partial least square regression.

**GWAS for the case study at group level**

To compare with the genes selected by SmCCNet, we implemented a GWAS for the associations between BMI and the 265 277 imputed and LD-pruned SNPs, which are the same input as SmCCNet. GWAS was conducted via PLINK (version 1.9) using the following bash code:

| #! /bin/bash  for i in {1..22}  do  plink --noweb --file chr${i}_geno_input --double-id --allow-no-sex --assoc --pheno BMI.txt --adjust --out chr${i}_GWAS_result &  done |
| --- |

We looped over the genotype data of 22 autosomes and used the flag ‘--noweb’ to skip web connection, ‘--double-id’ to ignore family IDs, ‘--allow-no-sex’ to allow no sex info, and ‘--assoc’ to compute regression statistics and Wald test. The phenotype BMI was adjusted for sex, age, age square, camera system, and four ancestry axes for population structure. The P-values from Wald test (Supp. Figure 1) were corrected for multiple testing based on Benjamini & Hochberg (1995) false discovery rate (FDR). We merged the results of all 22 autosomes and selected 155 SNPs with adjusted P-value $<0.05$. Subsequently, the same SNP-to-gene mapping as in ‘Data representation’ was applied to derive the list of genes whose SNPs are significantly associated with BMI. Out of the 155 significant SNPs, 87 were mapped to 95 genes.


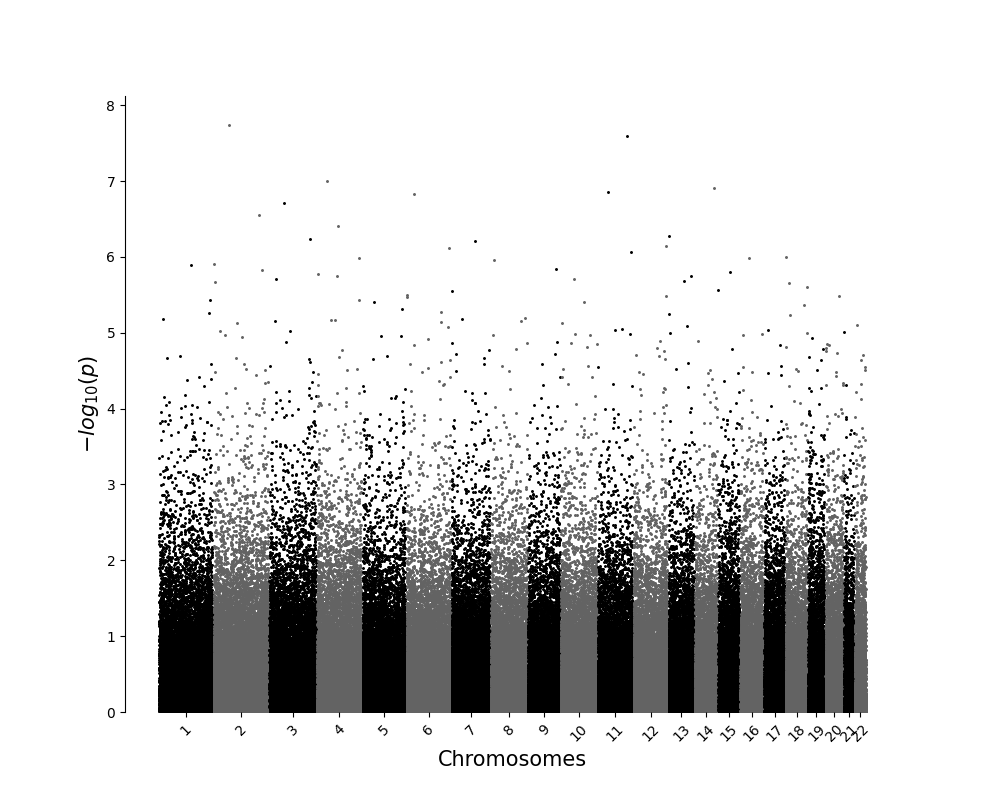


**Supp. Figure 1.** **Manhattan plot for the GWAS showing raw p-values of the associations between every SNP and BMI.**

**Enrichment of DisGeNET genes in SmCCNet genes**

We checked if the 873 BMI-associated genes from DisGeNET are significantly over-represented in the 278 BMI-related genes from SmCCNet than in the background of all 19 430 protein-coding genes. It was done by the R function ‘phyper’ which runs a hypergeometric test (or one-tailed Fisher’s exact test).

| **Datasets** | **Method** | **P-value** | **ARI** | **Time** |
| --- | --- | --- | --- | --- |
| X | Kmeans | 0.0596 | 0.0046 | 7.41 sec |
|  | PCA-Kmeans | 1.26e-4 | 0.0107 | 2.52 sec |
| Y | Kmeans | 0.1040 | 0.0104 | 9.27 sec |
|  | PCA-Kmeans | 0.0973 | 0.0080 | 2.40 sec |
| X, Y | Kmeans | 0.1403 | 0.0083 | 29.91 sec |
|  | PCA-Kmeans | 5.82e-4 | 0.0169 | 3.73 sec |
|  | iCluster+ | 0.9767 | 3.4e-4 | 10.55 min |
|  | iClusterBayes | 0.0135 | 0.0094 | 7.60 min |
|  | Spectrum | 0.2639 | 0.0015 | 15.57 sec |
|  | SNF | 2.23e-23 | 0.1156 | 8.31 sec |
|  | SmCCNet-Spectrum | 3.12e-7 | 0.0183 | 33.20 min |
|  | SmCCNet-SNF | 7.89e-65 | 0.4283 | 33.09 min |
|  | SmCCNet-ISN-SC | 3.97e-63 | 0.3021 | 37.15 min |
|  | **netMUG** | 6.14e-98 | 1 | 37.16 min |

**Supp. Table 1.** Performance of all methods on simulated data. Four out of six Kmeans baseline models use a single view (X or Y). Two-view Kmeans models are based on the concatenation of X and Y. ‘SmCCNet-Spectrum’ and ‘SmCCNet-SNF’ are benchmark models with features selected by SmCCNet. ‘SmCCNet-ISN-SC’ differs from the proposed framework, netMUG, with only the clustering method.


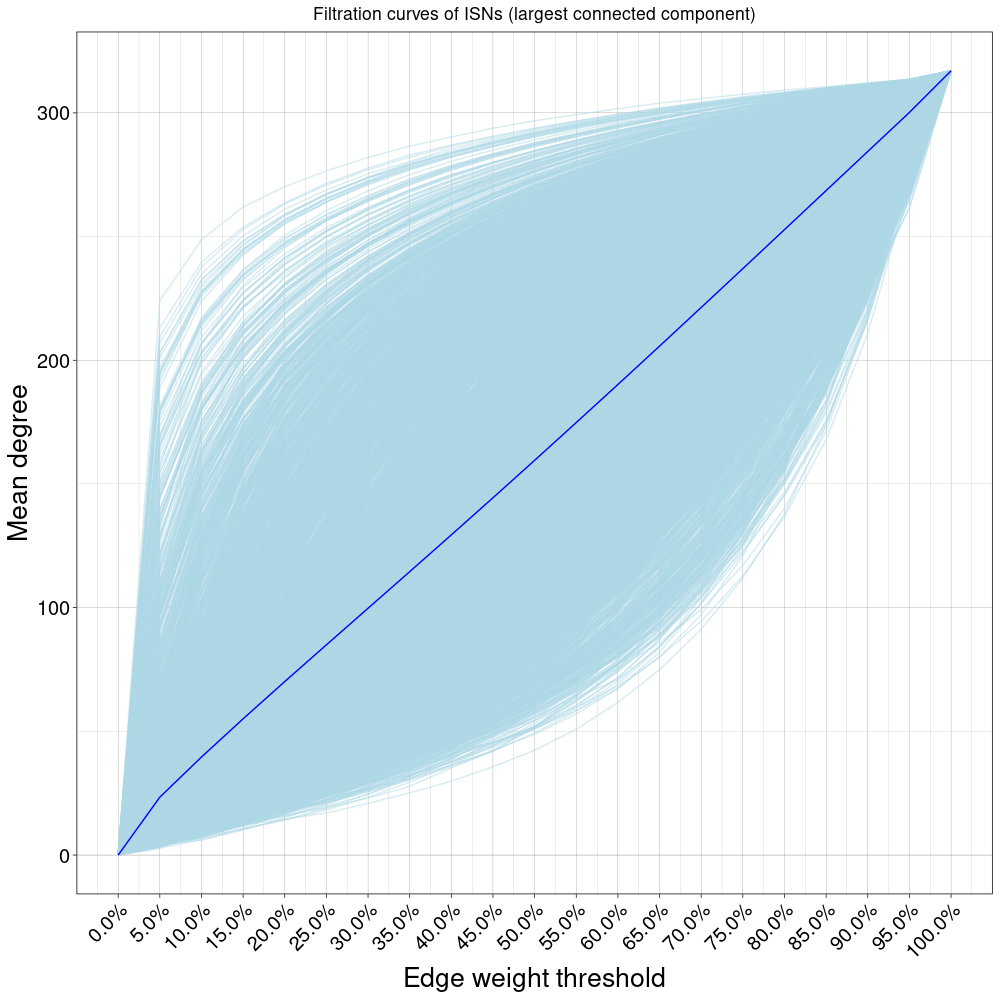


**Supp. Figure 2. Filtration curves of ISNs.** At each edge weight threshold, the mean degree of the largest connected component of the subgraph is computed as the function value. Every light blue line is a filtration curve representing an ISN or an individual. The thicker and darker line indicates the average of all filtration curves.
